# Are women with complications of an incomplete abortion more likely to be HIV infected than women without complications?

**DOI:** 10.1186/s12905-015-0237-7

**Published:** 2015-10-26

**Authors:** Carolyn Othieno, Joseph B. Babigumira, Barbra Richardson

**Affiliations:** Department of Health Services, Executive MPH Program, University of Washington, Seattle, WA USA; Department of Global Health, Global Medicines Program, University of Washington, Seattle, WA USA; Department of Pharmacy, Pharmaceutical Outcomes Research and Policy Program, University of Washington, Seattle, WA USA; Department of Biostatistics, University of Washington, Seattle, WA USA; P. O. Box 11679, Tacoma, WA 98409 USA

## Abstract

**Background:**

There is limited published evidence about the status of HIV among women who have had abortions or suffered from abortion complications. Understanding this connection is critical for building the evidence base and for guiding strategies to manage the sexual and reproductive health needs of women living with HIV.

The purpose of this study is to determine whether women who suffered incomplete abortion complications are more likely to be HIV infected than those without complications. We hypothesized that women with incomplete abortion complications have higher rates of HIV infection than women who attended clinic for other obstetric reasons.

**Methods:**

The analysis used a secondary dataset from a published case–control study that enrolled 1) 70 women at discharge after receiving in-patient care for complications resulting from induced abortion, and 2) 69 women (the comparison group) who visited the same hospital during the same time period for other obstetric needs. The primary outcome was seeking care for complications of incomplete abortion versus seeking care for other obstetric needs (dichotomous). The primary exposure variable was self-reported HIV status which was categorized into three groups: HIV positive, HIV negative, and HIV unknown. Unadjusted and adjusted associations between being in the abortion complications group, HIV status and other selected population characteristics were estimated using univariate and multivariate logistic regression.

**Results:**

Of 139 women enrolled in this study. Seventy (50.4 %) women had abortion complications and 69 (49.6 %) did not. Of the total study population, 18 (12.9 %) were HIV positive, 50 (36.0 %) were HIV negative, and the HIV status of 71 women (51.1 %) was unknown.

Compared to women who were HIV negative, women who were HIV positive had similar odds of being in the abortion complications group in both univariate and multivariate analyses (ρ =0.62 and ρ = 0.76). However, compared to HIV-negative women, those women who did not know their HIV status had greater odds of being in the abortion complications group (OR = 3.8, 95 % CI, 1.88, 8.20) in univariate analysis. After adjusting for potential confounding variables, the odds of being in the abortion complications group remained greater among women who did not know their HIV status compared to HIV-negative women (adjusted OR = 2.8, 95 % CI, 1.20, 6.54).

**Conclusions:**

This study points to the need for targeted interventions aimed at strengthening the delivery and coverage of HIV-testing programs for pregnant women and post-abortion care. In addition, more research is needed to better understand the relationships between unsafe abortion, abortion complications and unknown HIV status.

## Background

In the last decade, maternal deaths worldwide declined by 50 % [1]. Despite this decline, about 800 women die every day from preventable conditions associated with pregnancy and child birth. In 2010, 287,000 women died from conditions related to pregnancy and childbirth. And a large proportion (99 %) of these deaths occur in developing countries, with more than half in sub-Saharan Africa.

The maternal mortality rate in Uganda is among the highest in the world [2]. Three hundred and one maternal deaths occur for every 100,000 Ugandan women of reproductive age [2, 3]. It is known that complications during pregnancy and childbirth, such as severe hemorrhage, infection, hypertensive diseases, and unsafe abortion, are the leading causes of maternal mortality in the country [4].

### Unsafe abortion

**Fig. 1:**
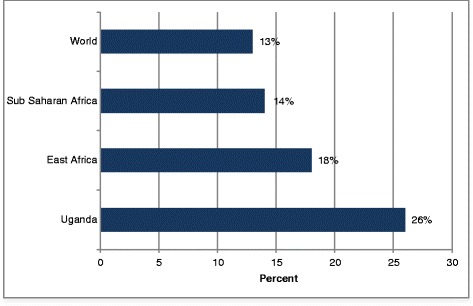
Percentage of maternal death resulting from unsafe abortion (2008) [5, 6]. The figure shows that the proportion of maternal deaths due to unsafe abortion in Uganda (26 %) far exceeds estimates for East Africa (18 %) and the World (13 %)

Unsafe abortion is a leading cause of maternal deaths in Uganda, accounting for 26 % of all maternal deaths in 2008 [5]. Figure 1 shows that the proportion of maternal deaths due to unsafe abortion in Uganda (26 %) far exceeds estimates for East Africa (18 %) and the World (13 %) [6]. Yet the law in Uganda prohibits abortion except for saving the life or the physical and mental health of the mother (Uganda, 1950). The demand for abortion in Uganda is high because of the high rate of unintended or unwanted pregnancies [3, 7]. One in two pregnancies in Uganda is unintended and about one in three of these pregnancies result in abortion [8]. In 2003, approximately 775,000 women, aged 15 to 49 years, had unintended pregnancies resulting in 297,000 abortions for an annual rate of 54 per 1000 [2]. That same year, approximately 85,000 (29 %) of the women who had abortions suffered from abortion-related complications [2].

Because abortion is illegal in Uganda except for reasons mentioned above, most abortions are carried out in a surreptitious manner by unskilled practitioners using unsafe techniques, putting women’s health and life at risk [9, 10]. Unsafe abortion results in lasting and devastating effects, including, incomplete abortions. Usually, these complications require hospitalization and specialized medical attention, which further strains the country’s limited health care resources [7].

In 2003, 1.5 % of Ugandan women aged 15 to 44 years, were hospitalized for abortion complications [7, 11]. But this is most likely an underestimate: for every woman hospitalized for an abortion complication, several others have had unsafe abortions and developed complications but don’t receive medical help or died before reaching the hospital [7]. For instance, in 2010, more than 50,000 women in need of medical care for abortion complications were untreated [12].

Unsafe abortion places a heavy burden on Uganda’s health care system. In 2009, the cumulative projected national expenditure on induced abortion was $64 million in societal costs [13] — more than 4 % of Uganda’s total annual health care expenditure of approximately $1.5 billion [14]. In addition, the annual cost of post- abortion care is about $13.9 million [12]. Therefore abortion is a significant economic and public health problem in the Uganda.

### Human Immunodeficiency Virus (HIV)

HIV remains a major public health challenge, contributing to maternal deaths in Uganda. HIV- infected women are eight times more likely to die due to pregnancy- related illness than women who are not infected with the virus [15, 16], and 24 % of pregnancy- related deaths are associated with HIV in sub- Saharan Africa [15, 16].

1.6 million people were living with HIV, more than 140,000 were infected and 63,000 died due to AIDS- related illness in Uganda, in 2013 [17]. HIV prevalence increased from 6.4 % in 2006, when rates were at their lowest to 7.2 % in 2011 [17]. This increase is attributable to new HIV infections and fewer AIDS- related deaths [18]. The epidemic disproportionately affects women in Uganda. In 2011, more than half (56 %) of adults living with HIV were women [19, 20]. HIV prevalence rates (8.3 %) are two times higher among women aged 15 to 49 than among men (6.1 %) of the same age [19]. Gender disparities in the country are driven by a range of factors including, biological vulnerability to HIV infection and limited access to HIV prevention and reproductive health services [21].

Fewer people are getting infected and dying from AIDS-related illness, but the number of new infections and AIDS- related deaths remain high. Uganda accounts for 6 % of all AIDS deaths in the sub-Saharan Africa [18], and is one of three countries responsible for almost half (48 %) all new HIV infections in the region [18]. At the same time, new HIV infections and AIDS-related deaths dropped by 13 % and 16 % between 2011 and 2013 in Uganda [17], as a result of scaling up antiretroviral therapy and HIV prevention efforts.

HIV testing and counselling (HTC) programs, including client-initiated, and provider-initiated HTC are key elements of Uganda’s national HIV response strategy [22]. It is known that knowledge of HIV status, has far-reaching influence on access to prevention, treatment and care services [23]. For example, in sub-Saharan Africa, 90 % of people who tested positive for HIV accessed antiretroviral therapy [18]. People who know their HIV-positive status are more likely to initiate early treatment and avoid risk of transmitting the virus to others [24]. And for those who are HIV- negative, knowing their status, motivates individuals to make informed behavioral choices in order to maintain their HIV- negative status [25].

### Unintended Pregnancy and Unsafe abortion in the context of HIV

The increasing interest in linking reproductive health and HIV has heightened the need for research exploring relationships between unintended pregnancy, unsafe abortion and HIV. Few studies have examined abortion in relation to HIV, and these studies suggest that HIV-positive women are as likely as other women to have abortions [26, 27], and that they resort to abortion for the same reasons as other women [28, 29].

Most studies have focused on HIV in relation to pregnancy and unintended pregnancy, and these studies show varying results. Some studies show that, HIV-positive women experience similar odds of unintended pregnancy as their HIV-negative counterparts [26, 30]. Other studies show that, HIV-positive women experience high rates of pregnancy [31], and unintended pregnancy. For example, HIV-positive women have rates of unintended pregnancy that are 51 to 90 % higher than HIV-negative women in Côte d’Ivoire, South Africa, and Uganda [32, 33]. This suggests that levels of abortion and complications from abortion may be equally as high among HIV- positive women.

Although these studies have found that HIV-positive women represent the majority of unintended pregnancies in Uganda, limited research exists on the prevalence of HIV among women who have had abortions and even less about women who have suffered from abortion complications. Understanding this connection is critical for building the evidence base for policy making, guiding strategies to manage HIV infection and unsafe abortion, and recognizing disparities in access to and delivery of post-abortion care by women with HIV.

In this paper, we describe the results of a study in which we compared the HIV-infection status of women with incomplete abortion complications to women seeking care at the same hospital for other obstetric needs. The purpose of this study is to determine whether women with complications of an incomplete abortion are more likely to be HIV infected than women without complications. We hypothesized that women with incomplete abortion complications might have higher rates of HIV infection than women who attended clinic for other obstetric reasons.

## Methods

### Design/study population/participants

We analyzed secondary data from a study assessing the economic burden associated with induced abortions in Uganda [13]. The data were collected from women who attended the Obstetrics and Gynecology unit of Mbarara University Teaching Hospital Uganda, between December 2009 and October 2010. Mbarara Hospital is a public funded regional referral hospital, serving a population of 2.5 million in 10 districts. The hospital has 350 beds and annual admissions of over 15,000 patients [34, 35].

The study population consisted of 139 women: 70 who enrolled at discharge after receiving in-patient care for complications resulting from induced abortion and 69 (the comparison group) who visited the same hospital during the same time period for other obstetric needs. The initial study used a continuous sampling strategy and included all women who met the inclusion criteria until the sample size was reached. All women enrolled in the study were above 18 years old and consented to the interview and follow-up [36].

### Variables

The primary outcome was seeking care for complications of incomplete abortion versus seeking care for other obstetric needs (dichotomous). The primary exposure variable was self-reported HIV status which was categorized into three groups: HIV positive, HIV negative, and HIV unknown.

Potential confounding variables included age, education, marital status, age of partner, education of partner, number of pregnancies, number of children, self-reported health status, and working status.

### Statistical analysis

All data were analyzed using SPSS Version 19.0. To facilitate our statistical analysis, continuous variables, i.e., age, age of partner, number of pregnancies, and number of children, were converted into categorical groups. Age was recoded into two categories: 18 to 29 years and 30 to 50 years. Age of partner was recoded into three categories: 18 to 35 years, 36 to 55 years and unknown. Number of pregnancies was recoded into three categories: 1 to 2 pregnancies, 3 to 4 pregnancies, and 4 to 10 pregnancies. Number of children was recoded into three categories: 0 children, 1 to 3 children and 4 to 8 children. Categorical variables, i.e., education, education of partner, marital and health status, were recoded from their original groups into more logical categories for our analysis. We developed the coding by “combining” or “collapsing” categories when the number of responses in one category was either too small or too large. Education was collapsed into four categories: (no education, primary school, secondary school, and higher than secondary school). Marital status was collapsed into two categories: (ever married and never married). Education of partner was collapsed into five categories; (no education, primary school, secondary school, higher than secondary school, and don’t know). Self-reported health status was collapsed into three categories: (excellent, good, and average/poor).

To control for outliers and maintain the full sample size, we winsorized age, education, partner age, partner education, number of children, and number of pregnancies using the last value carried forward method to impute missing and extreme values.

Univariate logistic regression was used to determine the independent association between abortion complications and each variable unadjusted for other risk factors. We used backward stepwise elimination to identify variables to include in the multivariate logistic model. All independent variables showing an association (*p* < 0.1) with abortion complications in the stepwise analysis were included in the multivariate logistic regression model. No variables included in the model were collinear.

### Power analysis

The study had 70 subjects with abortion complications and 69 without (controls). Assuming a 2-sided test with Type I error probability of 0.05, an overall rate of HIV infection of 13 % in the study, and a difference in the proportion of HIV infected among those with and without abortion complications between 10 and 20 %, the study had between 32 and 91 % power to detect such differences.

### Ethical review

The Institutional Review Board (IRB) of University of Washington waived the need for IRB approval.

### Consent statement

The Institutional Review Board determined that informed consent was not required. Since the study was a secondary data research, information for the analysis could not be linked back to individual subjects. Participants from the original study could not be individually identified or recognized.

## Results

### Baseline characteristics of study population

Table 1 presents the demographic characteristics of the study population. A total of 139 women were enrolled in this study. Seventy (50.4 %) women had abortion complications and 69 (49.6 %) did not. Of the total study population, 18 (12.9 %) were HIV positive, 50 (36.0 %) were HIV negative, and the HIV status of 71 women (51.1 %) was unknown. More than half (56.1 %) of the women were under the age of 29 years, and the majority (77.7 %) of the women had ever been married.

**Table 1:** Demographic characteristics of the study population by presence or absence of abortion complications

Characteristics	Overall	Abortion complications	No abortion complications
		*N* = 70 (50.4)	*N* = 69 (49.6)
HIV status			
Positive	18 (12.9)	8 (11.4)	10 (14.5)
Unknown	50 (36.0)	35 (50.0)	15 (21.7)
Negative	71 (51.1)	27 (38.6)	44 (63.8)
Age			
≤29 years	78 (56.1)	40 (57.1)	38 (55.1)
>29 years	61 (43.9)	30 (42.9)	31 (44.9)
Age of partner			
≤35 years	43 (30.9)	24 (34.3)	19 (27.5)
>35 years	32 (23.0)	13 (18.6)	19 (27.5)
Unknown	64 (46.1)	33 (47.1)	31 (44.9)
Education			
No schooling	21 (15.1)	9 (12.9)	12 (17.4)
Primary	53 (38.1)	32 (45.7)	21 (30.4)
Secondary	45 (32.4)	20 (28.6)	25 (36.2)
More than secondary	20 (14.4)	9 (12.9)	11 (15.9)
Education of partner			
No schooling	23 (16.5)	11 (15.7)	12 (17.4)
Primary	45 (32.4)	21 (30.0)	24 (34.8)
Secondary	35 (25.2)	15 (21.4)	20 (29.0)
More than secondary	21 (15.1)	11 (15.7)	10 (14.5)
Don’t know	15 (10.8)	12 (17.1)	3 (4.3)
Marital status			
Ever married	108 (77.7)	47 (67.1)	61 (88.4)
Never married	31 (22.3)	23 (32.9)	8 (11.6)
Number of children			
0	20 (14.4)	18 (25.7)	2 (2.9)
1-3	68 (48.9)	32 (45.7)	36 (52.2)
4-8	51 (36.7)	20 (28.6)	31 (44.9)
Number of pregnancies			
1-2	41 (29.5)	26 (37.1)	15 (21.7)
3-4	51 (36.7)	20 (28.6)	31 (44.9)
5-10	47 (33.8)	24 (34.3)	23 (33.3)
Health status			
Excellent	28 (20.1)	7 (10.0)	21 (30.4)
Good	83 (59.7)	46 (65.7)	37 (53.6)
Average/Poor	28 (20.1)	17 (24.3)	11 (15.9)
Work status			
Working	42 (30.2)	22 (31.4)	20 (29.0)
Not Working	97 (69.8)	48 (68.6)	49 (71.0)

*N* = 139. Seventy (50.4 %) women had abortion complications and 69 (49.6 %) did not. Of the total study population, 18 (12.9 %) were HIV positive, 50 (36.0 %) were HIV negative, and the HIV status of 71 women (51.1 %) was unknown. More than half (56.1 %) of the women were under the age of 29 years, and the majority (77.7 %) of the women had ever been married

### Correlates of abortion complications

Table 2 presents unadjusted and adjusted odds ratios for the association between being in the abortion complications group and selected population characteristics. In the univariate models, variables significantly associated with being in the abortion complications group were self-reported HIV status, marital status, number of children, and health status. Women who did not know their HIV status had an almost four times higher odds of being in the abortion complications group than women who were HIV negative (OR = 3.8, 95 % CI, 1.76, 8.23). Similarly, women who were never married had an almost four times higher odds of being in the abortion complications group compared to women who were ever married (OR = 3.73, 95 % CI, 1.53, 9.09). Having average to poor health compared to having excellent health increased the odds of being in the abortion complication group almost five-fold (OR = 4.64, 95 % CI, 1.48, 14.54). The odds of being in the abortion complications group were approxmately14 times greater for women with no children than for women with four or more children (OR = 13.95, 95 % CI, 2.92, 66.73). Age, age of partner, education, number of pregnancies, and work status were not significantly related to the odds of being in the abortion complications group.

**Table 2:** Univariate and multivariate logistic regression results showing the relationship between abortion complications, self-reported HIV status, and other characteristics

Characteristics	Crude OR (95 % CI)	*Ρ*-value	Adjusted OR (95 % CI)	*Ρ*-value
HIV status				
Positive	1.3 (0.46, 3.71)	.619	0.76 (0.23, 2.52)	.653
Unknown	3.8 (1.76, 8.23)	< .001	2.80 (1.20, 6.54)	.018
Negative (Ref)	1.00	_ _	_ _	_ _
Age				
≤29 years	1.1 (0.56,2.13)	.806	_ _	_ _
>29 years (Ref)	1.00	_ _	_ _	_ _
Age of partner				
≤35 years	1.19 (0.55, 2.58)	.666	_ _	_ _
>35 years	0.64 (2.27, 1.52)	.313	_ _	_ _
Unknown (Ref)	1.00	_ _	_ _	_ _
Education				
No schooling	0.92 (0.27,3.15)	.890	_ _	_ _
Primary	1.86 (0.66, 5.26)	.241	_ _	_ _
Secondary	0.98 (0.34, 2.82)	.967	_ _	_ _
More than Secondary (Ref)	1.00	_ _	_ _	_ _
Education of Partner				
No schooling	0.23 (0.05,1.03)	.055	_ _	_ _
Primary	0.22 (0.05,0.88)	.033	_ _	_ _
Secondary	0.19 (0.05, 0.78)	.022	_ _	_ _
More than Secondary	0.28 (0.06,1.27)	.098	_ _	_ _
Don’t Know (Ref)	1.00	_ _	_ _	_ _
Marital status				
Ever married (Ref)	1.00	_ _	_ _	_ _
Never married	3.73 (1.53,9.09)	.004	_ _	_ _
Number of children				
0	13.95 (2.92, 66.73)	.001	17.49 (3.17, 96.5)	.001
1-3	1.38 (0.66, 2.88)	.394	1.74 (0.78, 3.90)	.177
4-8 (Ref)	1.00	_ _	_ _	_ _
Number of pregnancies				
1-2 (Ref)	1.00	_ _	_ _	_ _
3-4	0.37 (0.16, 0.87)	.022	_ _	_ _
5-10	0.60 (0.26, 1.42)	.245	_ _	_ _
Health status				
Excellent (Ref)	1.00	_ _	_ _	_ _
Good	3.73 (1.43, 9.73)	.007	4.86 (1.53, 15.44)	.007
Average/Poor	4.64 (1.48, 14.54)	.009	6.77 (1.67, 27.49)	.007
Work status				
Working	1.12 (0.54, 2.32)	.754	_ _	_ _
Not working (Ref)	1.00	_ _	_ _	_ _

Multivariate analysis showed independent associations between being in the abortion complications group and HIV, and number of children and health status (Table 2). After adjusting for the three variables, women who did not know their HIV status maintained almost three-fold higher odds of being in the abortion complications group compared to HIV-negative women (aOR = 2.80, 95 % CI, 1.20, 6.54). The odds of being in the abortion complications group were over 17 times greater for women with no children compared to women with four or more children (aOR = 17.49, 95 % CI, 3.17, 96.5). Women with average to poor health had almost seven times greater odds of being in the abortion complications group than women with excellent health (aOR = 6.77, 95 % CI, 1.67, 27.49) and women with good health had almost five times greater odds of being in the abortion complications group compared to women with excellent health (aOR = 4.86, 95 % 95 % CI, 1.53, 15.44).

## Discussion

In this study, we assessed the relationship between abortion complications and self-reported HIV status. We hypothesized that women with incomplete abortion complications would have higher rates of HIV infection than women who attended clinic for other obstetric reasons; however, the findings of this study failed to support the hypothesis. The results of this study indicate that women seeking care for incomplete abortion complications had similar rates of HIV infection as women seeking care for other obstetric reasons. We found that HIV-positive and HIV-negative women had similar odds of having abortion complications.

One of the most striking findings was that, compared to HIV-negative women, women with unknown HIV status were at an increased risk of having abortion complications. We also found 36 % of women who had abortion complications did not know their HIV status. This finding suggests that despite efforts to promote prenatal HIV counseling and testing in Uganda, many women are still not being tested [37, 38].

Our study has some limitations. A major weakness of our study is the limited power to see differences between HIV-positive and negative women because of a small number of HIV-positive (18) women in our study population. A plausible explanation for the low number of HIV-positive women, could be that some women infected with HIV in our study population, do not know that they are infected. In Uganda, one of seven women aged 15–49 know their HIV status [3], and only two of five women of the same age had an HIV- test recently [3]. These findings emphasize the need to expand opportunities for HIV testing.

Another limitation of our study is that, our sample was not a probabilistic sample, as a result the sample may suffer from selection bias which limits the generalizability of our study findings. Additionally, a cross sectional, case–control study design cannot provide evidence of temporal relationship between exposure and outcome because the exposure and outcome are assessed simultaneously. That is to say that, even if a researcher determines that there is an association between exposure and outcome, there is generally no evidence to suggest that the exposure caused the outcome.

Given that a cross sectional study estimates prevalence instead of incidence, it does not account for subjects who acquire the outcome but die before the study. Consequently there may be a bias in data selection towards including subjects with more survivorship. Cross sectional studies are also susceptible to bias due to low response and misclassification due to recall bias. In this case, bias due to low response and misclassification may be introduced because of the secondary data analysis. The initial study (our data source) used self-reports to collect information on exposure status. Generally, the reliability and validity of self-reports may be questionable [39]. In this case, truthfulness and accuracy of our self-reported HIV data may be compromised because some health risks, such as HIV, are so sensitive that respondents may not want to accurately report them [40].

Given these limitations, these data must be interpreted with caution: the study generalizability is limited by the sample size and sampled population, which consists of data on women who received medical care from a specific hospital (Mbarara University Hospital) in western Uganda.

The current findings add to the growing body of literature on unsafe abortion in Uganda and are useful for generating hypothesis and identifying associations that can be more thoroughly investigated in future studies.

## Conclusions

Women seeking care for incomplete abortion complications had a similar rate of HIV infection compared to women who attended clinic for other obstetric reasons. However, women with unknown HIV status were significantly more likely to seek care for abortion complications, independent of other potential risk factors. This indicates that women most at risk for abortion complications are not being reached by the existing HIV testing interventions [41]. This study adds to the body of knowledge around the links between sexual and reproductive health and HIV. In addition, the results highlight the need expand the coverage of existing HIV testing interventions. More broadly, research is needed to determine further linkages between unknown HIV status and abortion complications.
